# Achieving large and nonvolatile tunable magnetoresistance in organic spin valves using electronic phase separated manganites

**DOI:** 10.1038/s41467-019-11827-0

**Published:** 2019-08-28

**Authors:** Wenting Yang, Qian Shi, Tian Miao, Qiang Li, Peng Cai, Hao Liu, Hanxuan Lin, Yu Bai, Yinyan Zhu, Yang Yu, Lina Deng, Wenbin Wang, Lifeng Yin, Dali Sun, X.-G. Zhang, Jian Shen

**Affiliations:** 1 State Key Laboratory of Surface Physics and Department of Physics, Fudan University, 200433 Shanghai, China; 2 Institute for Nanoelectronics Devices and Quantum Computing, Fudan University, 200433 Shanghai, China; 3 Department of Physics, North Carolina State University, Raleigh, NC 27695 USA; 4 Department of Physics and the Quantum Theory Project, University of Florida, Gainesville, FL 32611 USA; 5 Collaborative Innovation Center of Advanced Microstructures, Nanjing University, 210093 Nanjing, China

**Keywords:** Spintronics, Electronic and spintronic devices

## Abstract

Tailoring molecular spinterface between novel magnetic materials and organic semiconductors offers promise to achieve high spin injection efficiency. Yet it has been challenging to achieve simultaneously a high and nonvolatile control of magnetoresistance effect in organic spintronic devices. To date, the largest magnetoresistance (~300% at *T* = 10 K) has been reached in tris-(8-hydroxyquinoline) aluminum (Alq_3_)-based organic spin valves (OSVs) using La_0.67_Sr_0.33_MnO_3_ as a magnetic electrode. Here we demonstrate that one type of perovskite manganites, i.e., a (La_2/3_Pr_1/3_)_5/8_Ca_3/8_MnO_3_ thin film with pronounced electronic phase separation (EPS), can be used in Alq_3_-based OSVs to achieve a large magnetoresistance (MR) up to 440% at *T* = 10 K and a typical electrical Hanle effect as the Hallmark of the spin injection. The contactless magnetic field-controlled EPS enables us to achieve a nonvolatile tunable MR response persisting up to 120 K. Our study suggests a new route to design high performance multifunctional OSV devices using electronic phase separated manganites.

## Abstract (web summary)

Organic materials hold great potential of for spintronic applications. Here the authors show electronic phase dependent magnetoresistance (MR) effect in LPCMO/Alq_3_/Co junctions with large MR up to 440% at 10 K as well as electrical Hanle effect as the Hallmark of the spin injection.

## Introduction

Efficient spin injection from a ferromagnetic (FM) material into a nonmagnetic semiconductor is highly desirable for spin-based computing concepts such as spin transistors and spin storage/memory devices^1–4^. It is challenging due to conductivity mismatch between the FM contact and the semiconductor^5–7^ that greatly suppresses the spin injection efficiency. Inserting a high-quality tunneling barrier/spin filterer at the FM/semiconductor interface^8^ will improve the spin injection efficiency whereas a sophisticated epitaxial layer growth is required making it difficult to be compatible with other heterostructure systems^9–11^. The discovery of MR effect through an organic semiconductor (OSEC) medium between two FM contacts, namely organic spin valves (OSVs)^12–14^, suggests a new route to enhance the spin injection efficiency into semiconductors: The formation of OSEC/FM interface (so-called: spinterface^15–21^) offers a promising spin-filtering effect to enhance the spin polarization of FM metals leading to a large MR effect (up to 300%) at low temperature^17,22,23^. While most recent OSV studies focus on optimizing the MR performance by tailoring the OSEC/FM spinterface and exploring the multi-functionalities of OSVs by utilizing photovoltaic response in OSECs^9,19,24–28^, the influence of the FM contacts on the MR effect has not been emphasized.

In general, FM materials with high spin polarization are favorable for the FM contacts in the OSVs. In this regard, the half metallic perovskite manganites with near 100% spin polarization would be the most popular candidates. In spite of a large number of members in manganites family, La_0.67_Sr_0.33_MnO_3_ (LSMO) has been almost the only material used in OSVs^10,14,22,29,30^. This is probably because most other manganite films have pronounced electronic phase separation (EPS) featured by coexistence of ferromagnetic metallic (FMM) and non-ferromagnetic insulating phases, which would presumably lower the spin polarization and are thus considered as non-favorable materials for spin injection.

Here we demonstrate that by substituting the LSMO with a (La_2/3_Pr_1/3_)_5/8_Ca_3/8_MnO_3_ (LPCMO) film, a surprisingly large MR effect up to 440% is achieved in a conventional OSV geometry. Benefited from such a large magnetoresistance, a signature of electrical Hanle effect is successfully observed in the LPCMO-based OSV device, unambitiously showing the electrical spin injection and detection in the Alq_3_ layer. The LPCMO system has been well known for its pronounced EPS between the ferromagnetic metallic (FMM) and antiferromagnetic charge ordered insulating (COI) phases^31^. We show that magnetic field induced modulation of the FMM and COI phase in the LPCMO thin film leads to a tunable MR effect up to 120 K above which the phase separation disappears. Our results open a new pathway of designing high performance organic spintronic devices by utilizing the electronic phase separation of the complex manganites, thus bridging the gap between organic spintronics and complex manganites materials.

## Results

### Device structure and characterizations of LPCMO thin film

**Fig. 1:**
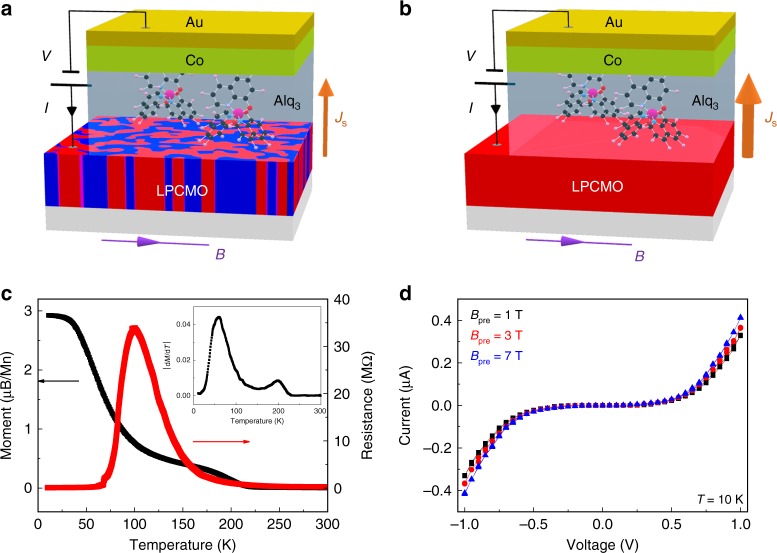
Schematic structure and transport properties of the OSVs using complex manganites electrode. **a**, **b** The illustration of EPS-modulated spin current injection in the LPCMO-OSVs under the co-existed FM/COI phase (**a**) and fully FM phase (**b**) of the LPCMO thin film, respectively. **c** The temperature dependence of magnetization (*M* vs. *T*) and resistance (*R* vs. *T*) of the LPCMO thin film. The inset shows the temperature dependence of $|dM/dT|$. **d** *I–V* characteristics of the LPCMO-OSV device at 10 K as a function of pre-set magnetic field (i.e., 1 T, 3 T, and 7 T)

We demonstrate EPS-modulated large MR response in a vertical-junction molecular spin valve device consisting of a 60-nm-thick Alq_3_ (tris-(8-hydroxyquinoline) aluminum, Aldrich) molecule thin film sandwiched between a 60-nm-thick LPCMO film epitaxially grown on the SrTiO_3_ substrate and a 10-nm-thick cobalt layer with Au capping. A schematic diagram of the fabricated OSV structure is shown in Fig. 1a, b (see Methods). The Alq_3_ films were used because it is the well-tested molecular material for constituting the organic/molecular spintronic devices. It provides a large spin filtering effect via the Alq_3_/ferromagnet spinterface as evidenced by two-photon photoemission spectroscopies^19^. The LPCMO thin film was selected since the high-quality LPCMO film grown by pulsed laser deposition exhibits a large-scale electronic phase separation that can be controlled by applying a pre-set field (*B*_pre_) or varying the temperature^31,32^. The LPCMO thin film is also suitable for an efficient spin injection owing to its half-metallic nature that would significantly suppress the conductivity mismatch between organic molecules and oxide surface. In support of this assertion, the spin polarization of LPCMO is calculated from the tunneling magnetoresistance (TMR) response in a LPCMO/Al_2_O_3_/Co devices using the Jullière model (i.e., ${\mathrm\it {TMR}} = \frac{{2P_{{\mathrm{LPCMO}}}P_{{\mathrm{Co}}}}}{{1 - P_{{\mathrm{LPCMO}}}P_{{\mathrm{Co}}}}}$)^33^. The obtained largest value of TMR is 93% at 10 K. Considering *P*_Co_ = 34% from the literature report^34^, we estimate that the *P*_LPCMO_ is ~93% at 10 K (see Supplementary Fig. 1 and Supplementary Note 1). Note that the application of the Jullière model here is questionable due to the large down-spin channel resistance of the LPCMO electrode, which is neglected by the Jullière model and tends to lower the estimated spin polarization. The actual value of *P*_LPCMO_ would be even higher.

The phase competition between FMM and COI domains in the LPCMO thin film^35–37^ is characterized by both temperature-dependent resistivity (*R* vs. *T*) and magnetization (*M* vs. *T*) measurements, as shown in Fig. 1c (please also see Supplementary Figs. 2 and 3). These two plots present a typical FMM-to-COI interconversion in the LPCMO thin film as a function of temperature^38,39^: When the temperature decreases, the paramagnetic (PM) phase is replaced by the COI phase at *T*_CO_ ~ 200 K, as revealed by the sharp increase of the thin film resistance. This transition is consistent with the onset of ferromagnetism as presented in the *M* vs. *T* and the derivative peak of $|dM/dT|$ vs. *T* curve (inset of Fig. 1c) at the same temperature. The short-range FMM phase domains grow with decreasing temperature and percolate to form conducting channels at the insulator-to-metal transition temperature (~100 K). The long-range ferromagnetic order forms at Curie temperature (*T*_c_ ~ 59 K) when most of COI phase domains are melt into the FMM phase.

Figure. 1d shows the typical nonlinear current-voltage (*I–V*) curves of the LPCMO-based OSV device at 10 K, which exhibit similar characteristics to those of a conventional LSMO/Alq_3_/Co OSV^14^. All *I–V* curves were measured at 1 T after the following magnetic field history: (1) a pre-set magnetic field, *B*_pre_, was applied along the in-plane direction; (2) The field was reduced to zero and then increased to 1 T. This procedure was repeated for higher *B*_pre_ without any demagnetization process in between. The conductance (resistance) increases (decreases) with increasing *B*_pre_, suggesting that the percolation of FMM phase in the LPCMO bottom electrode reduces the overall device resistance. For comparison, the LPCMO bottom electrode is replaced by La_0.67_Sr_0.33_MnO_3_ to form a LSMO-OSV. *I–V* curves of the LSMO-OSV do not exhibit any measurable dependence on *B*_pre_ (see Supplementary Fig. 4).

### Large MR effect in the LPCMO-OSV device

**Fig. 2:**
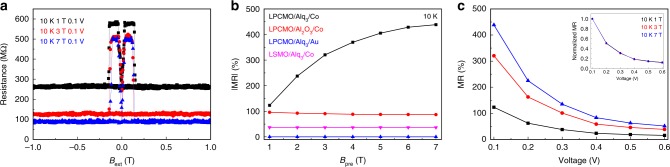
EPS-modulated MR response in the LPCMO-OSV device. **a** *MR*(*B*_ext_) loops of the LPCMO-OSV device under three pre-set magnetic field strength (i.e., 1 T, 3 T, and 7 T). MR measurements were taken at *T* = 10 K by applying a voltage bias *V* = 0.1 V. **b** The obtained $|MR|$ values from the LPCMO/Alq_3_/Co, LPCMO/Al_2_O_3_/Co, LPCMO/Alq_3_/Au, and LSMO/Alq_3_/Co devices as a function of pre-set magnetic field (*B*_pre_). **c** Bias dependence of MR values in the LPCMO-OSV device under the pre-set magnetic field of 1 T, 3 T, and 7 T, respectively. The inset shows the normalized *MR* vs. *V* curves in panel **c**

MR responses of the LPCMO-OSV as a function of pre-set magnetic field (*B*_pre_) are presented in Fig. 2a. The MR value was measured after the following procedure: (1) A chosen *B*_pre_ was applied along the in-plane direction and then reduced to zero; (2) A sweeping in-plane field was applied from 1 T to −1 T and back to 1 T. Upon sweeping field from 1 T to −1 T, the relative magnetization orientation of the two FM electrodes changes from parallel (P) to antiparallel (AP) and to parallel configurations, leading to a change of the device resistance (*R*) from *R*_P_ to *R*_AP_ and back to *R*_P_. A typical positive MR peak appears (*R*_P_ < *R*_AP_) corresponding well with the coercive fields of Co and LPCMO electrodes determined by SQUID measurements (see Supplementary Fig. 5). When the top Co electrode is replaced by a Au layer, there is no MR signal (see Supplementary Fig. 6). This implies that the observed MR response indeed originates from the switching of magnetization of the Co and LPCMO electrode, excluding the possibilities of the tunneling anisotropic magnetoresistance^35,40,41^ and organic magnetoresistance^42,43^. The abrupt change of device resistance between the parallel and antiparallel configuration indicates a high-quality device fabrication and well-defined interfaces between Alq_3_ molecules and ferromagnetic electrodes. We note that the sign of MR response is opposite to that in a LSMO OSV^14,17,22,29,30^ (see Supplementary Fig. 4). This indicates that the LPCMO electrode injects a spin current into the Alq_3_ layer with an opposite sign comparing to that from LSMO electrode. The sign of spin current can vary depending on the relative energy level alignment and interface between Alq_3_ and FM layer as manifested in previous reports^9,11,14,17,22^.

By applying *B*_pre_ = 1 T, a large MR signal close to +130% is obtained in the LPCMO-OSV device. Surprisingly, the MR amplitude increases with increasing *B*_pre_. We confirmed that this cannot be understood by simply counting the *B*_pre_ induced increase of FMM volume ratio, which would only reduce the device overall resistance but not MR of the device: Under a large preset magnetic field (e.g., *B*_pre_ = 7 T), both *R*_AP_ and *R*_P_ decrease but the decrease of *R*_P_ is much larger than *R*_AP_, resulting in a substantial enhancement of MR amplitude (up to ~440%) at high *B*_pre_. Such a large MR amplitude has not been observed in similar OSV devices using LSMO electrode^10,14,17,22,29,30^, suggesting a higher spin polarization injection from LPCMO electrode that is superior to the well-known LSMO electrode. The change of MR amplitude shows a non-linear increase as a function of *B*_pre_ and saturates at high preset magnetic fields (see Fig. 2b).

Three control experiments were performed to cross-check our results as summarized below (see Supplementary Fig. 4, 6, 7 and Fig. 2b): (1) A LSMO/Alq_3_/Co device exhibits a MR value of −37% at 10 K using the same growth condition and measurement procedures. But *B*_pre_ dependence of MR response was not observed; (2) A LPCMO/Alq_3_/Au device has no hysteretic MR response except for a weak anisotropic MR response from the LPCMO electrode; (3) A LPCMO/Al_2_O_3_/Co device exhibits a MR value of +30%. The device shows a negative *B*_pre_-dependent MR response, namely the MR ratio decreases as *B*_pre_ increases which is opposite to that in the LPCMO/Alq_3_/Co device. Hence we conclude that the formation of spinterface between Alq_3_ and LPCMO is critical to achieve a large and tunable MR response by *B*_pre_.

Bias dependence of MR response in the LPCMO-OSV device under different *B*_pre_ is plotted in Fig. 2c. The MR value increases with decreasing bias voltage, reaching a maximum at the lowest applied *V* = 0.1 V. A similar trend of bias dependence of the MR signal has been routinely observed in organic-based OSVs^11,14,22,29^ and in inorganic semiconductor/FM structures^44,45^, which might be attributed to the electric field dependence of the spin relaxation time/spin diffusion length in the molecule film^46,47^ or magnon excitation inside the FM electrodes^11,25,29,48^. Because the normalized $|MR|$ response shows no difference as a function of bias under different *B*_pre_ (inset of Fig. 2c), we can conclude that *B*_pre_ does not affect the electric field dependent spin relaxation time/spin diffusion length in Alq_3_ molecule film.

### Signature of electrical Hanle effect in the LPCMO-OSV device

**Fig. 3:**
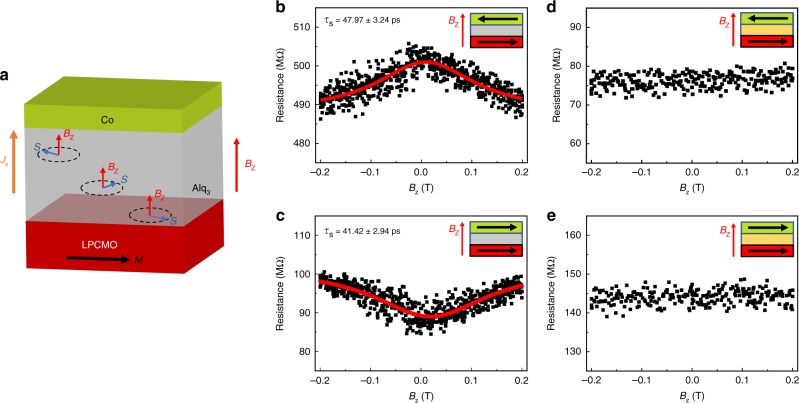
Signature of Hanle response in the LPCMO-OSV device. **a** Schematic illustrated Hanle effect in the LPCMO-OSV device. **b**, **c** Obtained electrical Hanle effect in the LPCMO-OSV device when a perpendicular magnetic field (*B*_z_) is applied to the device in the antiparallel (**b**) and parallel magnetic configuration (**c**), respectively. The red solid lines show the fitted plots using Eq. (1), from which the spin relaxation time, *τ*_s_ = 48.0 ± 3.2 ps and 41.4 ± 2.9 ps is extracted from the antiparallel and parallel configuration, respectively. The insets show the direction of magnetization of the two FM electrodes in two magnetic configurations. **d**, **e** No Hanle curve is observed in the magnetic tunnel junction device using the LPCMO electrode (LPCMO/Al_2_O_3_/Co) in two magnetic configurations, respectively. The Hanle measurements were performed at *T* = 10 K

To verify the spin-aligned carrier injection into the Alq_3_ layer, we conducted the electrical Hanle effect measurements^49^ in the LPCMO/Alq_3_/Co device at *T* = 10 K. An out-of-plane magnetic field, *B*_z_ was applied to the device (Fig. 3a) when the magnetization of the LPCMO and Co electrode are maintained in the antiparallel (Fig. 3b) or parallel configuration (Fig. 3c), respectively (see Methods). A typical Hanle effect is shown in the LPCMO-OSV device in both antiparallel and parallel configurations, which confirms the success of electrical spin injection and detection in the Alq_3_ interlayer. While using the same LPCMO electrode, the magnetic tunnel junction (i.e., LPCMO/Al_2_O_3_/Co) shows no Hanle effect (Fig. 3d, e), excluding the influence of the titled magnetization of FM electrodes on the device resistance. The applied perpendicular field (< ± 0.2 T) during the Hanle measurement does not title the magnetization of the LPCMO thin film (see Supplementary Fig. 8). The maintained magnetization states of two FM electrodes are also confirmed by repeating the in-plane MR loop after the Hanle measurement.

Here the spin lifetime, *τ*_s_ may be extracted by fitting measured Hanle effect with the equation^50,51^:

1 $$\Delta R_{{\mathrm{AP}}({\mathrm{P}})}\left( {B_{\mathrm{z}}} \right) = \Delta R_{{\mathrm{AP}}({\mathrm{P}})}(B_{\mathrm{z}} = 0)/(1 + (\omega _{\mathrm{L}}\tau _{\mathrm{s}})^2)$$

where *ω*_*L*_ is the Larmor frequency defined by $\omega _{\mathrm{L}} = g\mu _{\mathrm{B}}B_{\mathrm{z}}/\hbar$, the g-factor of holes in the Alq_3_ interlayer^14^ is equal to 2, *μ*_*B*_ is the Bohr magneton, ℏ is the reduced Planck’s constant, and $R_{{\mathrm{AP}}({\mathrm{P}})}(B_{\mathrm{z}} = 0)$ is the device resistance in the antiparallel and parallel configuration at *B*_z_ = 0T, respectively. By taking account of both magnetic configurations, we found the spin lifetime, *τ*_s_ ~ 45 ps for the injected spin ½ holes in the Alq_3_ OSV.

In contrast to the diffusion-dominated non-local Hanle measurement^52^, we found the change of device resistance in the antiparallel configuration is subtle (changing from ~500 MΩ to ~490 MΩ). It doesn’t decrease to the same level of the resistance as that in the parallel configuration (~90 MΩ), or vice versa. This indicates that the measured Hanle effect would be attributed to the partial spin precession under *B*_z_ when the spin carrier promptly transmits through the Alq_3_ interlayer^53^ due to the exchange-mediated spin transport^54,55^. Thus the obtained spin lifetime from the Hanle effect should be considered as the transit time, *τ*_transit_ or the lower bound of spin lifetime in the Alq_3_ interlayer.

It is the first time that the electrical Hanle effect is demonstrated in the Alq_3_-based OSVs. Due to the large in-plane magnetic anisotropy of the LPCMO electrode and the efficient spinterface, the large MR response up to 440% allows us to probe such a subtle Hanle effect in the LPCMO-OSV device, namely less than 4% of change in the device resistance at *B*_z_ = 200 mT that is two orders of magnitude smaller than its MR value. As a comparison, it is expected that the similar Hanle effect in the LSMO-based OSV device may be much smaller and beyond the instrument sensitivity (<0.4% if the MR value is ~40%^14^), probably accounting for the absence of Hanle effect in most Alq_3_-based OSVs. In addition, the short transit time in the Alq_3_ layer (*τ*_transit_ ~ 45 ps) would result in a very broad Hanle effect in the need of the perpendicular magnetic field at least up to 100–200 mT which is inaccessible in most OSVs using the soft LSMO electrode.

### Electronic phase separation correlated MR effect

The observed results suggest that the electronic phase separation (EPS) inside the LPCMO film plays a key role in the unusually large and tunable MR effect. To unravel the correlation between EPS and the observed MR response, a variable-temperature, high field magnetic force microscope (MFM) was used to visualize the FMM-to-COI phase interconversion as a function of *B*_pre_ and temperature (see Methods).

**Fig. 4:**
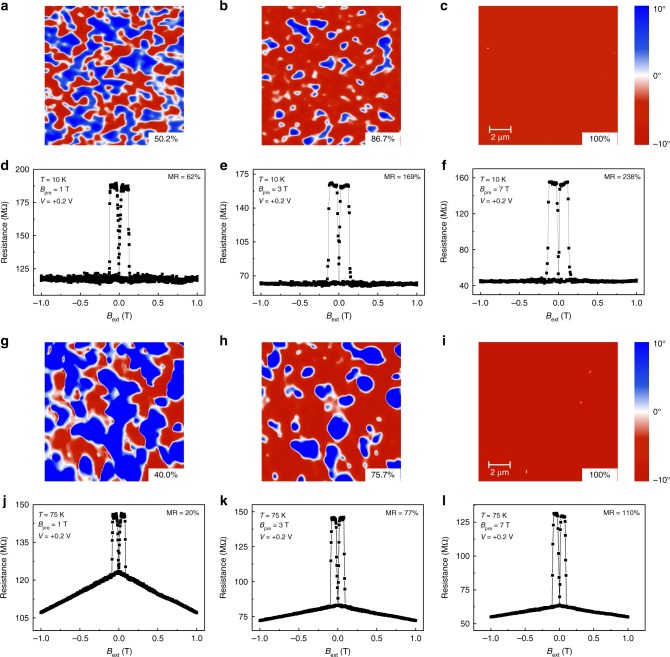
Temperature and pre-set magnetic field controlled EPS and MR response. **a**–**c** The obtained EPS image by magnetic force microscope in the LPCMO thin film at 10 K after applying the pre-set magnetic field, *B*_pre_ = 1 T, 3 T, and 7 T, respectively. The red area in the MFM image represents the FMM phase, while the blue area refers to the COI phase. All the MFM images were taken at a constant magnetic field (*B*_ext_ = 1 T) after withdrawing *B*_pre_. **d**–**f** The corresponded MR response in the LPCMO-OSV device under the same condition. The obtained EPS image (**g**–**i**) and *MR*(*B*_ext_) loops (**j**–**l**) at 75 K after applying the pre-set magnetic field, *B*_pre_ = 1 T, 3 T, and 7 T, respectively

Figure 4a, c show the MFM images of the LPCMO bottom electrode and Fig. 4d, f show the MR response of the same LPCMO-based OSV device measured at 10 K after application of *B*_pre_ = 1 T, 3 T, and 7 T, respectively. MFM images were recorded following the same procedure used in the MR measurements. All the MFM images were taken at a constant magnetic field (*B*_ext_ = 1 T) after withdrawing *B*_pre_. It is clear that increasing *B*_pre_ reduces the area fraction of the COI phase (blue area) and eventually all the COI phase domains transit into the FMM phase at a high *B*_pre_. This is consistent with the increase of magnetization as *B*_pre_ increases (see Supplementary Fig. 9). The area fraction of the FMM phase is calculated to be 50.2%, 86.7%, and 100% at *B*_pre_ = 1 T, 3 T, and 7 T, respectively. In addition, the comparison of MFM images, the data analysis and magnetic hysteresis loops of the LPCMO film, the LPCMO/Alq_3_(1 nm) and LPCMO/Al_2_O_3_(1 nm) indicates that the phase boundary and FMM volume fraction remain virtually unchanged with the Alq_3_ or Al_2_O_3_ capping layer (see Supplementary Figs. 10, 11, 12). The MR response increases accordingly with increasing FMM area fraction, as shown in Fig. 4d, f.

**Fig. 5:**
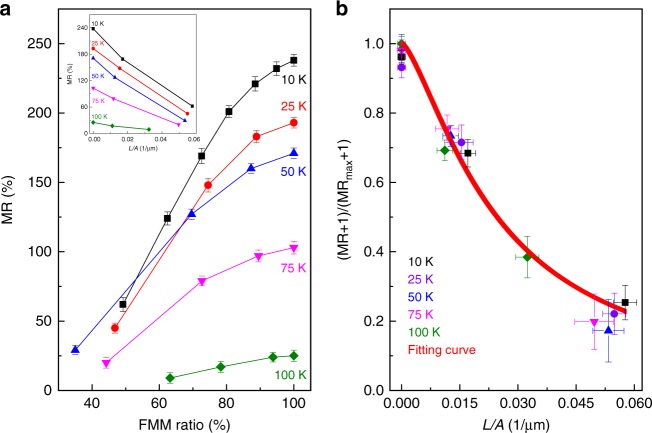
Nonlinear increase of MR response in LPCMO-OSV as a function of FMM ratio and phase boundary length per FMM area at different temperatures. **a** Obtained MR response as a function of FMM ratio controlled by applying different pre-set magnetic field (*B*_pre_). The inset shows the change of MR response as a function of the total length of phase boundary per FMM area (i.e., *L/A* ratio). The *L/A* ratios are calculated from the MFM images in Fig. 4 (i.e., FMM-fraction of red area; the total length of phase boundary per unit- fraction of white area per 10 μm×10 μm) at 10 K, 25 K, 50 K, 75 K, and 100 K after applying the pre-set magnetic field, *B*_pre_ = 1 T, 3 T, and 7 T, respectively. **b** Calculated $\frac{{{\mathrm\it{MR}} + 1}}{{{\mathrm\it{MR}}_{{\mathrm{max}}} + 1}}$ using the Eq. (2) as a function of *L/A* ratio, consistent with the experimental results in the inset of panel **a**. MR measurements were taken at *V* = 0.2 V. The vertical error bars in MR represent the nondeterminacy of the MR measurements and the fitting value of *MR*_max_, the lateral error bars in *L/A* represent the statistical uncertainty of the MFM measurements of *L* and *A*

Figure 4g to i show the MFM image of the LPCMO bottom electrode and Fig. 4j, l show the MR response of the same LPCMO-based OSV device measured at 75 K after application of *B*_pre_ = 1 T, 3 T, and 7 T, respectively. The MR response at 75 K again increases with *B*_pre_ induced increasing area fraction of the FMM phase. The maximum MR value obtained at 75 K (*B*_pre_ = 7T) is about two times smaller than that at 10 K, which is not surprising for OSV devices because the spin diffusion length in Alq_3_ thin film decreases with increasing temperature^11,15^. The high field MR response (near linear slope) becomes more pronounced at 75 K than at 10 K due to the decreased overall hysteric MR response in the device. The relations between the obtained MR values and the area fraction of the FMM phase at different temperature are summarized in Fig. 5a. The MR value increases nonlinearly as a function of the area fraction of the FMM phase.

This behavior cannot be attributed to the simple enlargement of the device cross-section due to the expansion of FMM phase, which would only cause the MR response to be unchanged or reduced as observed in the LPCMO/Al_2_O_3_/Co device (i.e., equivalent to the case by applying a larger current flow at a higher bias voltage, see Supplementary Fig. 5). Instead, we plot *MR* as a function of *L/A*, where *L* is the total length of the phase boundary and *A* is the area fraction of the FMM phase (inset of Fig. 5a). We find that the result can be approximately described by the relationship,

2 $$\frac{{{\mathrm\it{MR}} + 1}}{{{\mathrm\it{MR}}_{{\mathrm{max}}} + 1}} \approx \frac{1}{{1 + \alpha \frac{L}{A}}}$$

The fitting parameter, *α* = 35.6 ± 3.5 μm is obtained throughout the entire temperature range, indicating the key role of boundary formation between FMM and COI phase on the observed tunable MR response (see Fig. 5b).

### Two-current model with spin-flip scattering

**Fig. 6:**
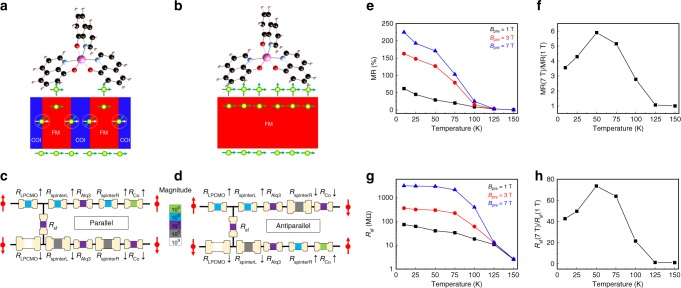
Theoretical model of EPS-modulated spin injection. **a**–**b** Illustrated EPS-modulated spin current into the Alq_3_ space layer under the co-existed FM/COI phase and fully FM phase, respectively. The yellow dots indicate the injected spin-aligned carriers and the green arrows indicate the direction of spin polarization. The spin-aligned carriers undergo a spin-flip scattering at the FM-COI boundary through the LPCMO thin film. It results in a reduced spin current into the Alq_3_ molecules compared to that in a fully FM phase of the LPCMO film in the absence of FM/COI boundary. **c**, **d** Equivalent circuit models in the parallel and antiparallel configurations under the co-existed FM/COI phase, respectively. Resistors are schematic with color codes, not drawn to accurate values. **e** Obtained MR values of the LPCMO-OSV device as a function of temperature and pre-set magnetic field. **f** normalized *MR*(7 T)/*MR*(1 T) as a function of temperature. **g**, **h** Calculated *R*_sf_ and normalized *R*_sf_(7 T)/*R*_sf_(1 T) from the proposed theoretical model as a function of temperature, consistent with the experimental results in panels **e** and **f**

The two-current model is the basis of all theoretical models of giant and tunneling magnetoresistance^56^. The celebrated Jullière model is in fact a special limit of the two-current model in which the electrode resistance is set to zero and the tunnel barrier is assumed to be spin-independent (see Supplementary Note 2 and Supplementary Fig. 13). In order to explicitly include the effect of the electrode resistance, whose change under the magnetic field is essential for understanding the nonlinearly enhanced MR effect under *B*_pre_ in the LPCMO-OSV device, and the strong spin-dependence of the spinterface resistance, we cannot use the simple Jullière model (see Fig. 6a, b). Here we start from the original two-current model that can capture all the key features of the experimental results including the LPCMO electrode resistance and interfacial spin filtering effect at the metal/molecule interface: The spin up (↑) and spin down (↓) carrier undergoes two different paths through LPCMO electrode, LPCMO/Alq_3_ spinterface^21^, Alq_3_ bulk layer, Alq_3_/Co spinterface^19^, and Co electrode from which a spin-dependent resistance is generated (*R*_*i*↑(↓)_, *i* denotes the different layer), respectively (see Fig. 6c, d). The interfacial spin filtering effect^17^ induced spin-dependent resistance at LPCMO/Alq_3_ and Alq_3_/Co spinterface are represented by $R_{{\mathrm{spinterL}}({\mathrm{R}}) \downarrow }$, with the corresponding up-spin channel spinterface resistances set to zero. In our model, we propose that the formation of EPS in the LPCMO film produces a significantly large spin-orbit coupling at the FMM/COI boundary because of the inversion symmetry breaking^57^. It leads to an additional spin-flip scattering between the spin-up and spin-down channel that can be equivalently represented as *R*_sf_ in the circuit (which effectively provides a short circuit due to the large difference in resistance between the two spin channels). The spin-flip scattering rate is approximately proportional to the total area of the phase boundary. Thus *R*_sf_ is an effective resistance due to spin-flip scattering and should be inversely proportional to the area of the FMM/COI phase boundary. The modified two-current model yields the following (see Supplementary Note 3):

For the device resistance in the parallel configuration (*R*_p_):

3 $$R_{\mathrm{P}} \approx R_{{\mathrm{LPCMO}} \uparrow } + R_{{\mathrm{Alq}}3} + R_{{\mathrm{Co}} \uparrow } \approx R_{{\mathrm{Alq}}3};$$

For the device resistance in the antiparallel configuration (*R*_AP_):

4 $$	{R_{{\mathrm{AP}}} \approx} \\ 	 {\frac{{R_{\mathrm{LPCMO} \downarrow} }\left( {R_{{\mathrm{Alq}}3} + R_{{\mathrm{spinterL}} \downarrow }} \right)\left( {R_{{\mathrm{Alq}}3} + R_{{\mathrm{spinterR}} \downarrow }} \right) + R_{{\mathrm{sf}}}\left( {R_{{\mathrm{LPCMO}} \downarrow } + R_{{\mathrm{Alq}}3} + R_{{\mathrm{spinterL}} \downarrow }} \right)\left( {R_{{\mathrm{Co}} \downarrow } + R_{{\mathrm{Alq}}3} + R_{{\mathrm{spinterR}} \downarrow }} \right)}{{R_{{\mathrm{LPCMO}} \downarrow }\left( {2R_{{\mathrm{Alq}}3} + R_{{\mathrm{spinterL}} \downarrow } + R_{{\mathrm{spinterR}} \downarrow }} \right) + R_{{\mathrm{sf}}}\left( {R_{{\mathrm{LPCMO}} \downarrow } + 2R_{{\mathrm{Alq}}3} + R_{{\mathrm{spinterL}} \downarrow } + R_{{\mathrm{spinterR}} \downarrow }} \right)}}}$$

where $R_{{\mathrm{Alq}}3}$ is the spin independent junction resistance of the Alq_3_ bulk, $R_{{\mathrm{LPCMO}} \uparrow }$ and $R_{{\mathrm{LPCMO}} \downarrow }$ is the resistance in the majority (spin-up) and minority (spin-down) channel of LPCMO film, respectively. $R_{{\mathrm{spinterL}} \downarrow }$ and $R_{{\mathrm{spinterR}} \downarrow }$ are the resistance arising from the spin-filtering effect in the down-spin channel on the LPCMO (L) side and the Co (R) side, respectively.

With zero or a small *B*_pre_, the area of FMM/COI boundaries inside LPCMO thin film is large because of strong EPS. A strong spin-flip scattering at the boundary causes *R*_sf_ to be close to zero and randomize the spin-up and spin-down channel, resulting in a significantly decreased spin polarization degree via LPCMO film. Hence there will be no net spin polarization current transmitting through the LPCMO film and thus MR value is small (see Fig. 6a).

By applying a large *B*_pre_, the FMM/COI boundaries diminish because of the percolation of FMM phases (Fig. 6b). The effective cross-section of the device increases, causing *R*_Alq3_, *R*_spinterL↓_,*R*_spinterR↓_, and *R*_LPCMOL↓_ to decrease inversely proportionally to the effective cross section area of the FMM phase. Presumably, the MR effect should remain unchanged if all the resistances decrease with the same proportion. However, the boundary induced spin flip scattering is greatly suppressed due to diminished FMM/COI phase mixing, i.e., *R*_sf_ becomes significantly larger ( → ∞). The increase of *R*_sf_ compensates the overall reduction of *R*_LPCMO↓_ in Eq. (4). It leads to a slightly reduced *R*_AP_ in the antiparallel configuration, compared to the greatly reduced *R*_P_ as shown in Fig. 2a. Thus Δ*R*(*B*_pre_) increases as a function of *B*_pre_, making the MR response increase dramatically. We note that the absence of the enhanced MR response in the LPCMO/Al_2_O_3_/Co device suggests that a highly polarized spin filtering effect (i.e., a large *R*_spinterL(R)↓_) at the LPCMO/Alq_3_ spinterface is required to realize the observed results (see Supplementary Fig. 7). A nonlinear increase of spin filtering resistance as a function of spin polarization degree from the LPCMO/Alq_3_ spinterface may also contribute to the overall MR response. Therefore, the observed large MR response can be understood as synergistic contributions from the tunable spin polarization degree at the FMM/COI boundary and spin polarization dependent spin filtering effect at the LPCMO/Alq_3_ spinterface. To connect Eq. (4) with the MR change as a function of FMM ratio and phase boundary length, we assume that *R*_sf_, *R*_LPCMO↓_, and *R*_spinterL(R)↓_ are larger than all other resistances, and $R_{{\mathrm{spinterL}}({\mathrm{R}}) \downarrow } \ll R_{{\mathrm{LPCMO}} \downarrow }$. The AP resistance is simplified to,

5 $$R_{{\mathrm{AP}}} \approx \frac{{R_{{\mathrm{sf}}}R_{{\mathrm{spinterR}} \downarrow }}}{{R_{{\mathrm{spinterL}} \downarrow } + R_{{\mathrm{spinterR}} \downarrow } + R_{{\mathrm{sf}}}}};$$

We further assume that $R_{{\mathrm{spinterL}}({\mathrm{R}}) \downarrow }\left( {B_{{\mathrm{pre}}}} \right) = R_{{\mathrm{spinterL}}({\mathrm{R}}) \downarrow }(0)/A$, $R_{{\mathrm{Alq}}3}\left( {B_{{\mathrm{pre}}}} \right) = R_{{\mathrm{Alq}}3}(0)/A$ where *A* is the FMM ratio, and *R*_sf_ = C/L where *L* is the total length of the phase boundary and *C* is a constant. The MR becomes,

6 $$\begin{array}{l}{\mathrm{MR}} + 1 = \frac{{R_{{\mathrm{AP}}}}}{{R_{\mathrm{P}}}} \approx \\ \frac{{CR_{{\mathrm{spinterL}} \downarrow }\left( 0 \right)}}{{R_{{\mathrm{Alq}}3}\left( 0 \right)\left[ {R_{{\mathrm{spinterL}} \downarrow }\left( 0 \right) + R_{{\mathrm{spinterR}} \downarrow }(0)} \right]\frac{L}{A} + R_{{\mathrm{Alq}}3}\left( 0 \right)C}} = \\ \frac{{MR_{{\mathrm{max}}} + 1}}{{1 + \frac{{R_{{\mathrm{spinterL}} \downarrow }\left( 0 \right) + R_{{\mathrm{spinterR}} \downarrow }(0)}}{C}\frac{L}{A}}};\end{array}$$

Comparing to Eq. (2), we find $\alpha = \frac{{R_{{\mathrm{spinterL}} \downarrow }\left( 0 \right) + R_{{\mathrm{spinterR}} \downarrow }(0)}}{C}$.

Our proposed model is further validated by the temperature dependence of MR response in the LPCMO-OSV device. Figure 6e shows the obtained MR values as a function of temperature under different *B*_pre_ at *V* = 0.2 V. In the presence of *B*_pre_, the MR response decreases monotonically with increasing temperature, and disappears above *T* ~ 150 K due to diminished magnetization of the LPCMO film (Fig. 1c). The ratio between *MR*(*B*_pre_ = 7 T) and *MR*(*B*_pre_ = 1 T) is plotted as a function of temperature in Fig. 6f. The *MR*(*B*_pre_ = 7 T)/*MR*(*B*_pre_ = 1 T) ratio shows a maximum around *T* = 50 K, which is consistent with the trend of $|dM/dT|$ vs. *T* plot in the inset of Fig. 1c. The experimentally obtained values of *MR*, *R*_P_, and *R*_AP_ at each temperature and *B*_pre_ are put into Eqs. (5) and (6), from which *R*_sf_ can be numerically calculated and is plotted in Fig. 6g. We found that the temperature-dependence of the ratio between *R*_sf_ at *B*_pre_ = 7 T and *B*_pre_ = 1 T (Fig. 6h) agrees well with that of the *MR*(*B*_pre_ = 7 T)/*MR*(*B*_pre_ = 1 T) ratio shown in Fig. 6f. This provides a compelling evidence that the boundary-induced spin-flip scattering (*R*_sf_) plays an essential role in determining the MR response in the LPCMO-OSV device.

## Discussion

We demonstrate that a large MR effect of ~440% can be created in Alq_3_ molecule-based spin valve device using a LPCMO thin film with pronounced EPS as the bottom electrode. The EPS state can be tuned by applying a *B*_pre_, allowing the contactless control of area fraction of the FMM phase in the bottom electrode. As a result, the MR response can be tuned by *B*_pre_ within six times difference in magnitude. Such an unusually large yet tunable MR effect is likely governed by a tunable spin-flip scattering at the boundaries between FMM and COI phases inside the LPCMO film and a large spin filtering effect at the molecule/oxide spinterface. The large MR effect enables us to demonstrate the first electrical Hanle effect in the Alq_3_-based OSV device as the success of spin injection and detection in the organic layer. The obtained spin lifetime (or the transit time) of electrically injected spin carriers (~45 ps) is surprisingly shorter than that of the bulk Alq_3_ layer despite its weak spin-orbit coupling, indicating the dominant role of the exchange-mediated spin transport. Our results demonstrate a novel approach for tuning MR response in organic spintronic devices via controlling EPS state in manganite electrodes. The proposed phase boundary-induced spin flip mechanism sheds light on understanding the origin of spin injection efficiency in the manganite films. Our study opens a new route towards logic switching for future high-performance molecular memory/storage devices.

## Methods

### Sample fabrication

LPCMO films (60 nm) were grown on SrTiO_3_ (100) substrates under the oxygen pressure of 8 × 10^−4^ Torr using pulsed laser deposition^58^. During the deposition, the film thickness was monitored by in-situ Reflection High Energy Electron Diffraction (RHEED). The LPCMO bottom electrodes were fabricated from the LPCMO thin films by wet photo-lithography^59^. After ultrasonic cleaning in alcohol and acetone, the LPCMO electrodes were immediately transferred into a vacuum chamber with a base pressure of 7.5 × 10^−11^ Torr. The Alq_3_ (99.995%, Aldrich) spacer layer (thickness: ~60 nm) was thermally evaporated at room temperature, followed by e-beam deposition of Co (10 nm)/Au (10 nm) top electrodes in a crossbar configuration using a shadow mask. The substrate was kept at 280 K during the top electrode deposition process to suppress the interdiffusion between the top electrode and the Alq_3_ layer^10^. The thickness of each layer was monitored by a quartz thickness monitor which had been calibrated by a profilometer. The active device area was about 20 × 120 μm.

### Magnetization and magnetoresistance measurements

Magnetization measurements were carried out using a Quantum Design superconducting quantum interference device system (SQUID). Magnetic fields were applied parallel to the device plane, which is the easy magnetization direction for both bottom and top magnetic electrodes.

Magnetoresistance measurements were carried out using a Quantum Design Physical Property Measurement System (PPMS) combined with a Keithley 2400 source meter. The magnetic fields were applied along the in-plane direction of the device. The MR is defined as *MR* = (*R*_antiparallel_−*R*_parallel_)/*R*_parallel_, where *R*_antiparallel_ (*R*_parallel_) is the device resistance in the antiparallel (parallel) magnetic configuration of the two ferromagnetic electrodes. The device resistance was measured by two-point probe method.

The MR response is controlled by three factors: temperature (*T*), pre-set magnetic field (*B*_pre_), and applied bias (*V*). Prior to measurements at each chosen temperature (e.g., 10 K, 25 K, 50 K, 75 K, 100 K, 125 K, and 150 K), the device was always set to room temperature following by cooling under zero magnetic field. After the chosen temperature was reached, *B*_pre_ (1 T, 2 T, 3 T, 4 T, 5 T, 6 T, and 7 T) was first applied to the device and then removed allowing us to control the area fraction of the FMM phase in the LPCMO bottom electrode. A voltage bias (e.g., *V* = 0.1 V, 0.2 V, 0.3 V, 0.4 V, 0.5 V, and 0.6 V) was applied to the device from which the resistance of device was recorded using a LabVIEW program. The MR response was measured by sweeping the magnetic field continuously from 1 T to −1 T and back to 1 T.

### Hanle measurements

The Hanle effect measurements were taken at *T* = 10 K by applying a voltage bias *V* = 0.1 V in the PPMS system. For the Hanle measurement in the parallel configuration, the MR loop is firstly measured along the in-plane magnetic field direction under the *B*_pre_ = 7 T to achieve the largest MR value in this device (~440%). After the MR loop measurement, the in-plane magnetic field is directly sweeping back to zero in order to maintain a ‘parallel’ configuration of two magnetic layers of the device (i.e., the low resistance state). Second, the device is rotated by 90 degrees to apply a perpendicular magnetic field (i.e., *B*_z_ is sweeping from +0.2 T to −0.2 T) to measure the Hanle effect in this parallel configuration of the device.

For the Hanle effect in the antiparallel configuration, the MR loop is firstly measured along the in-plane field direction to achieve the largest MR. From the *B*_in-plane_ = +1 T, the magnetic field is sweeping down to *B*_in-plane_ = −30 mT and then reducing to zero to achieve an antiparallel configuration of two ferromagnetic layers (i.e., the high resistance state). Then the device is rotated by 90 degrees to apply the perpendicular magnetic field (i.e., *B*_z_ is sweeping from +0.2 T to −0.2 T) to perform the Hanle measurement.

After finishing the Hanle measurement, the device is rotated back to the in-plane field geometry to make sure that the same MR loop is repeated after applying the perpendicular magnetic field.

### Magnetic force microscope measurements

MFM images were acquired from the same LPCMO thin film which was later used as the bottom electrode for LPCMO-OSV device. The MFM measurements were carried out in the PPMS System using Attocube scanning probe microscope following the same procedure for magnetoresistance measurements. For measurements at different temperatures, the temperature was first raised to room temperature at which the LPCMO becomes paramagnetic, and then cooled under zero field to a chosen temperature for MFM measurements on the LPCMO thin film. Note that the temperature cycle does not change the global resistivity and magnetization of the LPCMO film at a chosen temperature, although the EPS pattern varies even at the same chosen temperature after different temperature cycle. All the MFM images were taken at a constant magnetic field (*B*_ext_ = 1 T) after withdrawing *B*_pre_. The calculated area fraction of the FMM phase based on the MFM images should thus be similar to that in the LPCMO bottom electrode of the device.

## Supplementary information

Supplementary Information

## Data Availability

The data that support the findings of this study are available from the corresponding authors upon reasonable request.
