# Supplementary material for: Achieving large and nonvolatile tunable magnetoresistance in organic spin valves using electronic phase separated manganites
Source: Nat Commun. 2019 Aug 28;10:3877. doi: 10.1038/s41467-019-11827-0 (PMC6713754; doi:10.1038/s41467-019-11827-0)
Supplement: Supplementary file 1 — Supplementary Information [file 41467_2019_11827_MOESM1_ESM.pdf]

Supplementary Information

Achieving large and nonvolatile tunable magnetoresistance in organic spin valves using electronic phase separated manganites

Yang et al.

Outline

Supplementary Figure 1. MR response in the LPCMO/Al_2O_3/Co device.
Supplementary Figure 2. Magnetic force microscope images of the LPCMO thin film at different *T* and B_{pre}.
Supplementary Figure 3. Exchange bias response in the LPCMO thin film as a function of AF-COI area at different temperatures.
Supplementary Figure 4. LSMO/Alq_3/Co/Au devices (LSMO-OSV).
Supplementary Figure 5. Magnetic hysteresis loops of the thin film and the device.
Supplementary Figure 6. LPCMO/Alq_3/Au devices.
Supplementary Figure 7. LPCMO/Al_2O_3/Co/Au devices.
Supplementary Figure 8. The out-of-plane magnetic hysteresis loop of the LPCMO thin film.
Supplementary Figure 9. Magnetic hysteresis loops of the LPCMO film along the in-plane direction at different *T* and B_{pre}.
Supplementary Figure 10. Temperature and pre-set magnetic field-controlled EPS.
Supplementary Figure 11. Statistical analysis of the phase boundary per unit and FMM.
Supplementary Figure 12. Comparison of magnetic hysteresis loops of the LPCMO, LPCMO/Alq_3(1nm) and LPCMO/Al_2O_3(1nm) thin film.
Supplementary Figure 13. The normalized effective spin polarization of the LPCMO/Alq_3 spinterface as a function of phase boundary length per FMM area at different temperatures.

Supplementary Note 1. The estimated spin polarization of the LPCMO thin film
Supplementary Note 2. Connection to the Jullière model
Supplementary Note 3. Two-current model with spin-flip scattering

Supplementary Figures

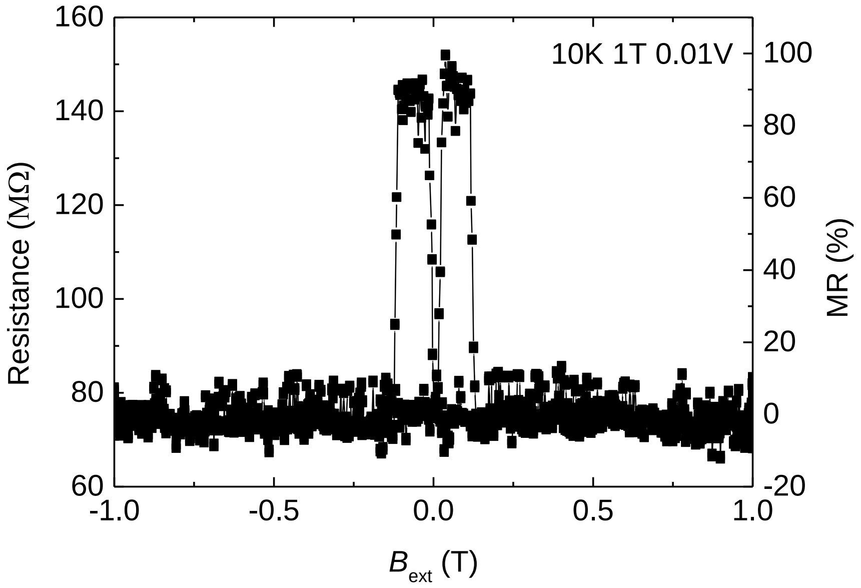

Supplementary Figure 1. MR response in the LPCMO/Al_2O_3/Co device. A *MR*(B_{ext}) loop of the LPCMO/Al_2O_3/Co device under the pre-set magnetic field of 1T. MR measurements were taken at T = 10K at the voltage bias V = 0.01V. The voltage bias is chosen to be close to zero in order to achieve the maximum TMR response.

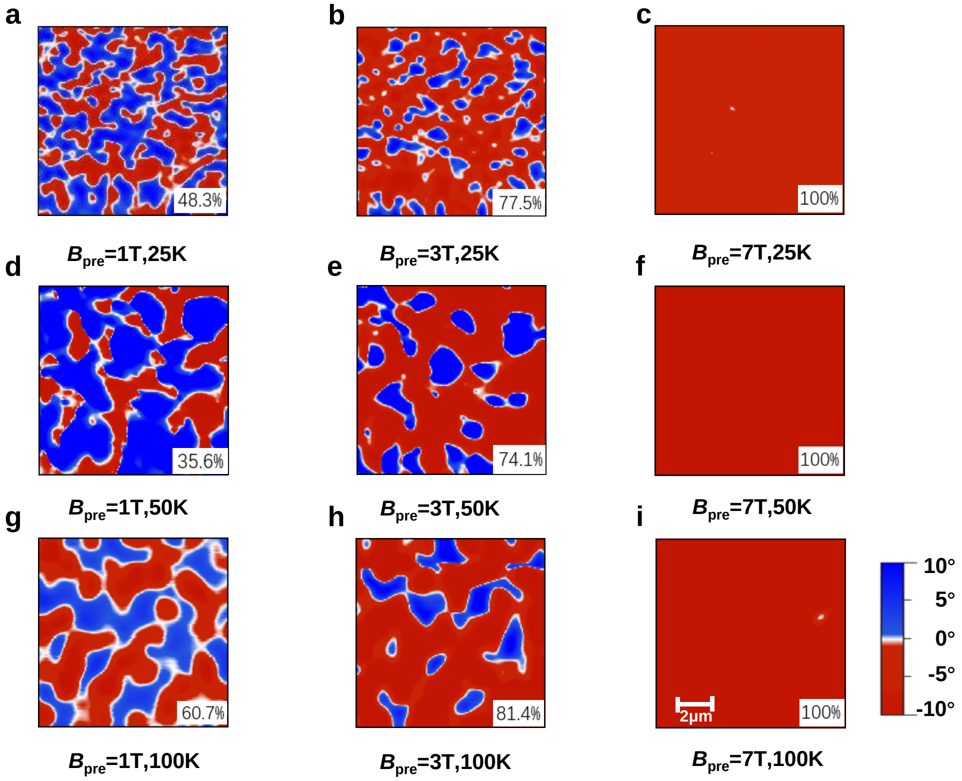

Supplementary Figure 2. **Magnetic force microscope images of the LPCMO thin film at different *T* and B_{pre}.** Measured MFM images at T = 25K **(a-c)**, 50K **(d-f)**, and 100K **(g-i)** under B_{pre} = 1T, 3T, 7T, respectively. All the MFM images were taken at a constant magnetic field (B_{ext}=1T) after withdrawing B_{pre}. Magnetic force microscope (MFM) images have been normalized to directly visualize the microscopic magnetic states of the LPCMO thin film. The red color region of the MFM image represents the FMM state, while the blue and white color refers to the COI state and boundary between FMM and COI phase, respectively. The calculated area fraction of the FMM phase has been shown at the corner of each image. The MFM measurements were carried out in the PPMS System using Attocube scanning probe microscope following the same procedure for MR measurements (see Methods).

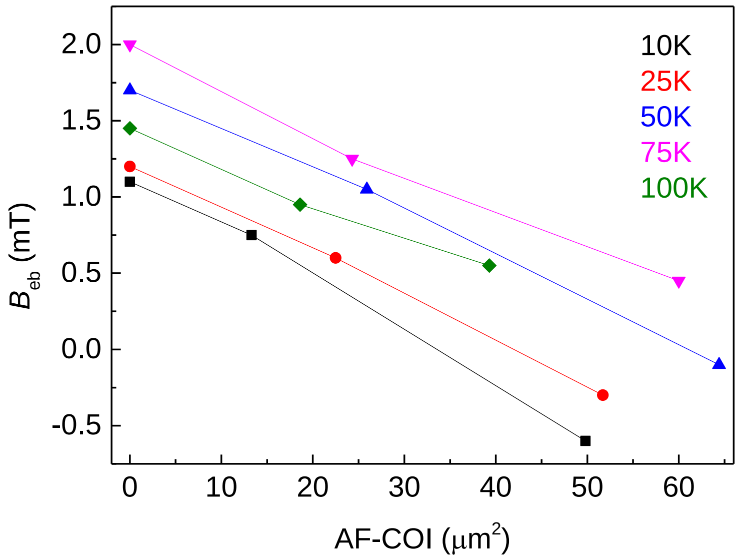

Supplementary Figure 3. Exchange bias response in the LPCMO thin film as a function of AF-COI area at different temperatures. AF-COI is controlled by applying different pre-set magnetic field (B_{pre}) before taking the SQUID measurement. The area fraction of the AF-COI phase is calculated from the MFM images in Figure.4 (i.e., AF-COI: fraction of blue area).

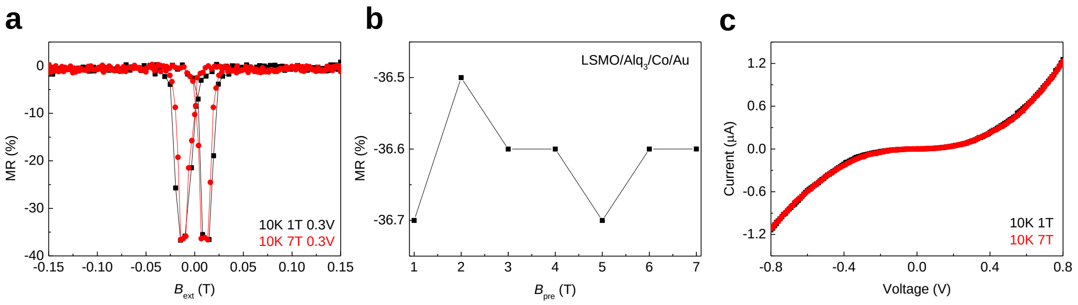

Supplementary Figure 4. **LSMO/Alq$_3$/Co/Au devices (LSMO-OSV). (a)** Measured $MR(B_{ext})$ loop of a LSMO (60 nm)/Alq$_3$ (60 nm)/Co (10 nm)/Au (10 nm) device under a bias voltage of V = 0.3V at T = 10K. The black (red) plot represents the MR loop under B_{pre} = 1T (7T). The negative sign of MR response is consistent with the literature reports elsewhere[1-5]. **(b)** B_{pre} dependence of obtained MR values in the LSMO-OSV device. **(c)** Nonlinear *I*-*V* curve of the LSMO-OSV device under B_{pre}=1T (black) and 7T (red) at 10K, respectively. Both MR values and *I*-*V* curves show no B_{pre} dependence.

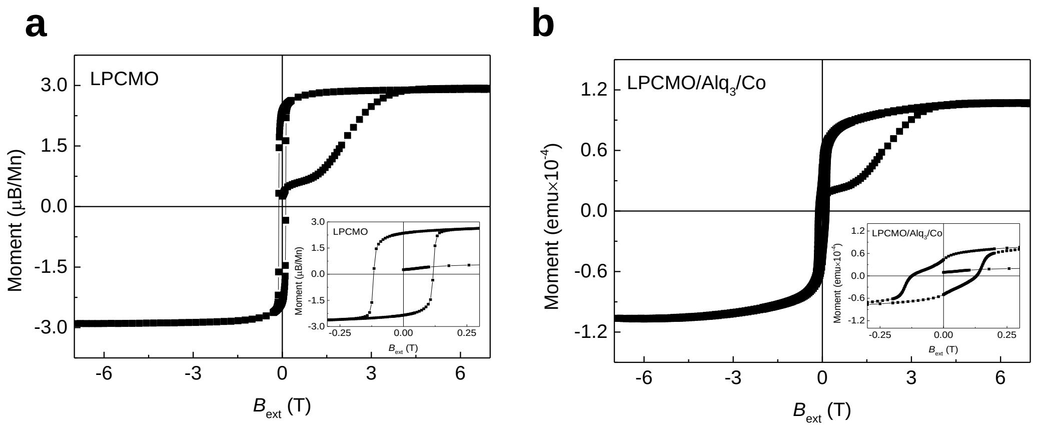

Supplementary Figure 5. **Magnetic hysteresis loops of the thin film and the device.** Measured the *M*-*B* loops of **(a)** the LPCMO film and **(b)** the LPCMO/Alq_3/Co device at T = 10K using SQUID magnetometry, respectively. The insets show the *M*-*B* loops between B = -300 mT to +300 mT, from which the coercive fields of LPCMO film and Cobalt electrode can be determined (B_c(LPCMO) ≈ 118 mT, B_c(Co) ≈ 30 mT). The obtained coercive fields are consistent with the switching fields of the MR loops.

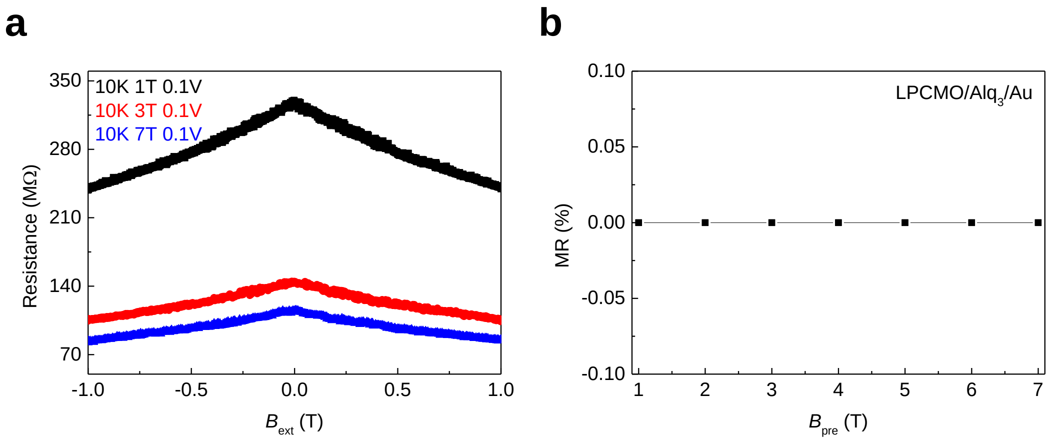

Supplementary Figure 6. LPCMO/Alq$_3$/Au devices. (a) Measured $MR(B_{ext})$ loop of a LPCMO (60 nm)/Alq$_3$ (60nm)/Au (10 nm) device as a function of B_{pre} at T = 10K by applying the same bias voltage of V = 0.1V, in which only one FM electrode (i.e., LPCMO) is used in the device configuration. No hysteretic MR response is observed except for a high-field anisotropic magnetoresistance originated from the LPCMO thin film. The absence of hysteretic MR response in the LPCMO/Alq$_3$/Au device unambiguously excludes the possibility of tunneling anisotropic magnetoresistance response in our LPCMO-OSV device[6-8]. **(b)** B_{pre} dependence of obtained MR values in the LPCMO/Alq$_3$/Au devices.

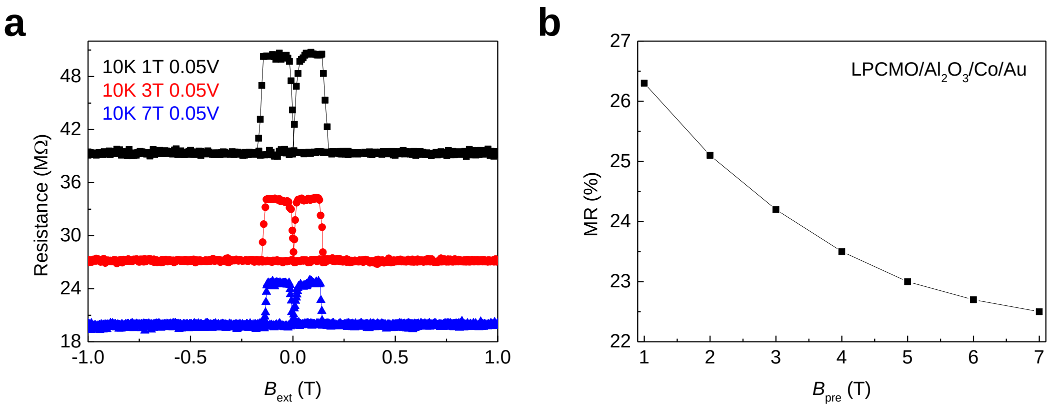

Supplementary Figure 7. LPCMO/Al_2O_3/Co/Au devices. (a) Measured $MR(B_{ext})$ loops of a LPCMO (60 nm)/Al_2O_3 (~1 nm)/Co (10 nm)/Au (10 nm) device as a function of B_{pre} at T = 10K by applying the same bias voltage of V = 0.05V. **(b)** B_{pre} dependence of MR values in the LPCMO/Al_2O_3/Co/Au device. MR values reduce monotonously with increasing $B_{pre,}$ which is in contrast with the increased MR values in the LPCMO-OSV device. This suggests that a highly polarized spin filtering effect at the LPCMO/Alq_3 spinterface is required to realize the observed large and tunable MR response.

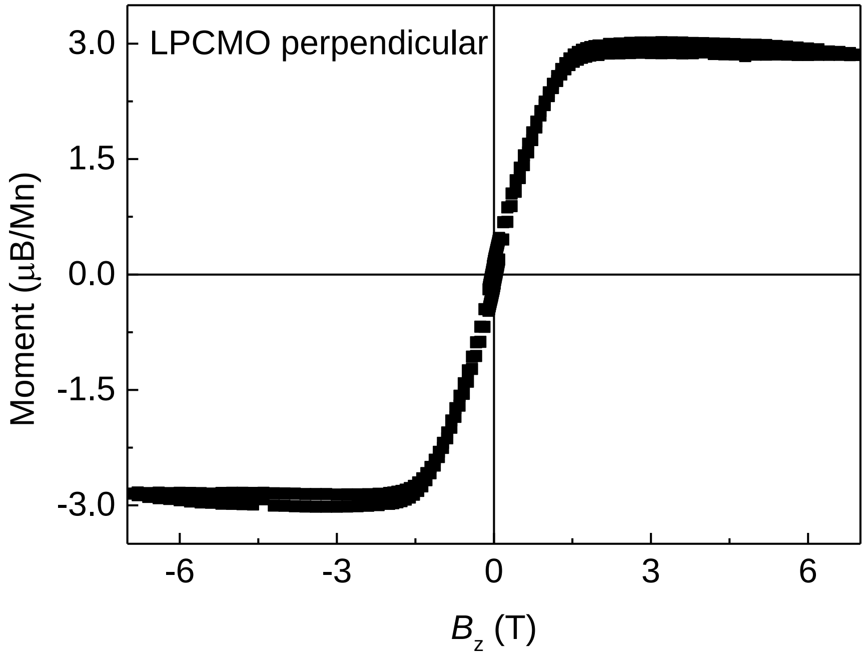

Supplementary Figure 8. The out-of-plane magnetic hysteresis loop of the LPCMO thin film. The hard-axis of magnetic anisotropy is along the out-of-plane direction. The applied perpendicular field (< ±0.2 T) during the Hanle measurement does not title the magnetization of the LPCMO thin film.

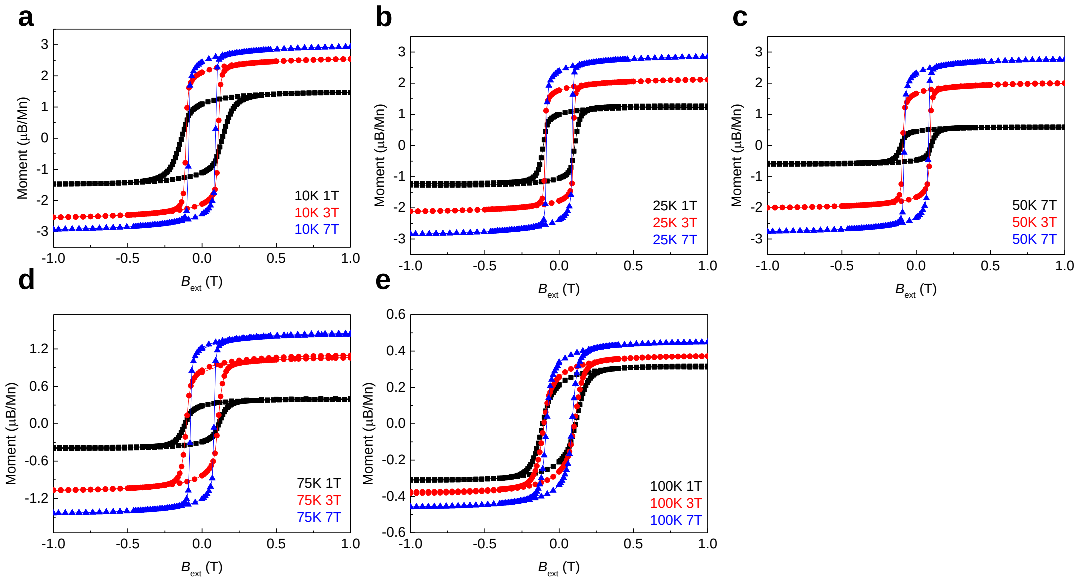

Supplementary Figure 9. Magnetic hysteresis loops of the LPCMO film along the in-plane direction at different *T* and $B_{\mathbf{pre.}}$ Measured *M-B* loops at *T* = 10K **(a)**, 25K **(b)**, 50K **(c)**, 75K **(d)**, and 100K **(e)**, respectively. The magnet moment of LPCMO film keeps increasing with increasing B_{pre}, indicating that the volume ratio of the percolated FMM phase increases with preset field, consistent with the MFM images in the Supplementary Fig. 2.

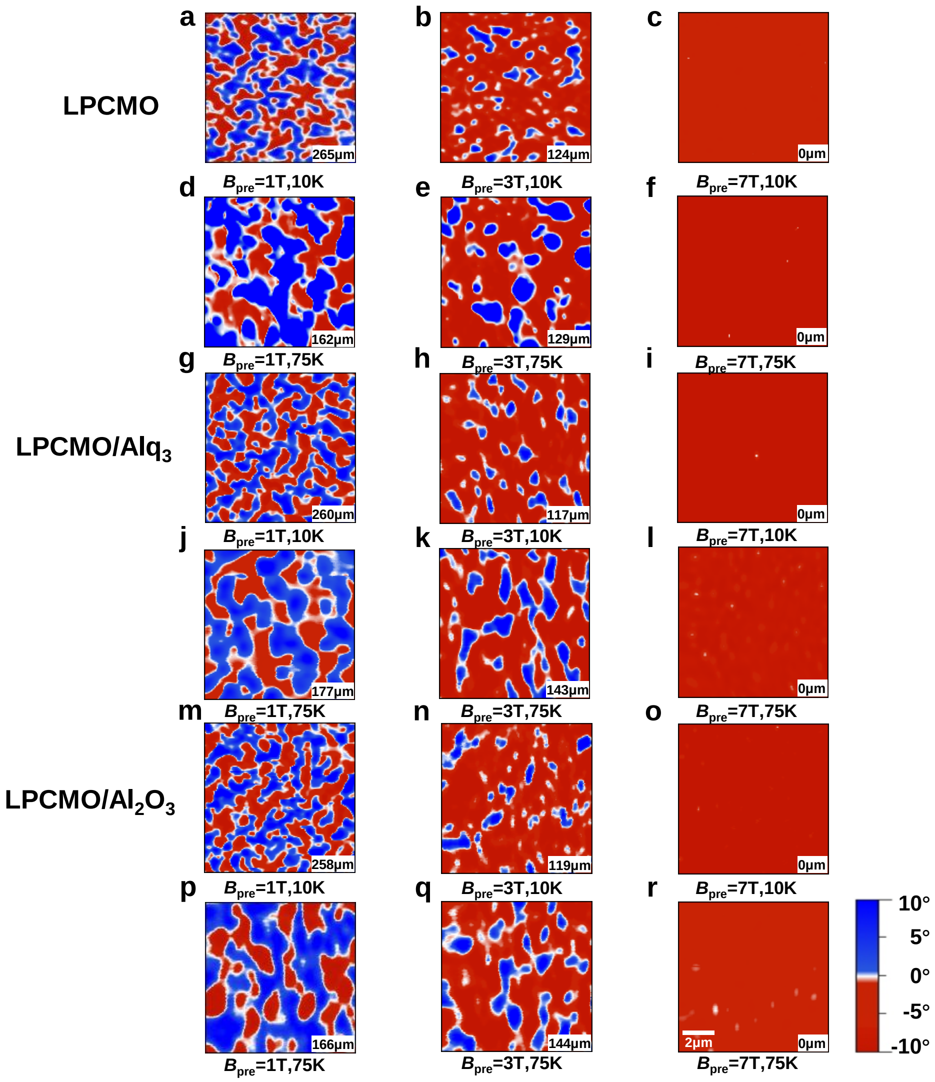

Supplementary Figure 10. Temperature and pre-set magnetic field-controlled EPS. The obtained EPS images by magnetic force microscope in the LPCMO thin film **(a-c** and **d-f)**, LPCMO/Alq_3(1nm) **(g-i** and **j-l)** and LPCMO/Al_2O_3(1nm) **(m-o** and **p-r)** at 10K and 75K after applying the pre-set magnetic field, B_{pre}=1T, 3T, and 7T, respectively. The red area in the MFM image represents the FMM phase, while the blue area refers to the COI phase. The white area is the phase boundary of FMM/COI. The perimeter of the white area is noted on the bottom right of each MFM image. All the MFM images were taken at a constant magnetic field (B_{ext}=1T) after withdrawing B_{pre}.

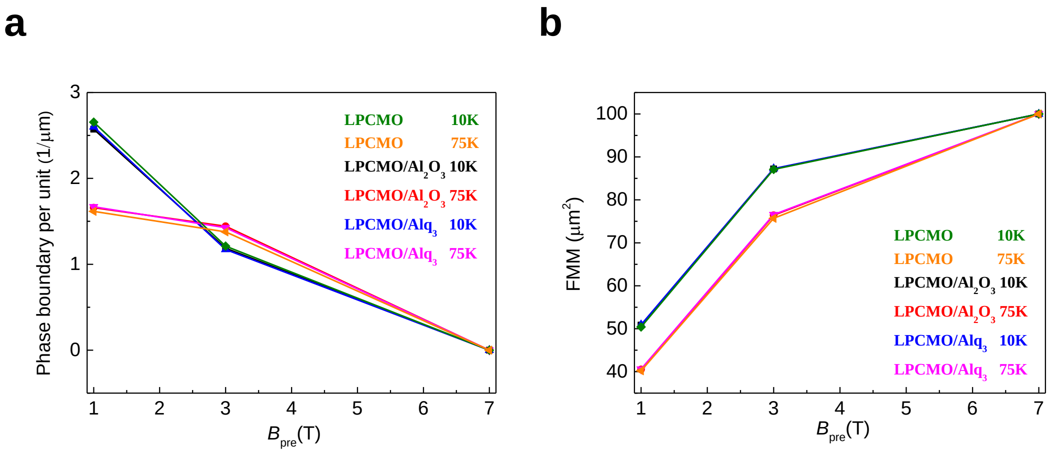

Supplementary Figure 11. Statistical analysis of the phase boundary per unit and FMM. (a) Bpre dependence of the total length of phase boundary per unit. **(b)** the area of FMM calculated from the MFM images of the LPCMO, LPCMO/Alq_3(1nm) and LPCMO/Al_2O_3(1nm) thin film at 10K and 75K, respectively.

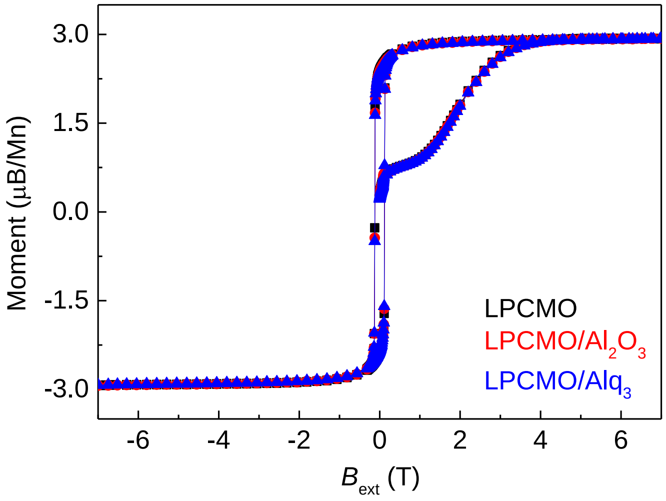

Supplementary Figure 12. Comparison of magnetic hysteresis loops of the LPCMO, LPCMO/Alq_3(1nm) and LPCMO/Al_2O_3(1nm) thin film. Measurements of magnetic hysteresis loops are taken at T = 10K.

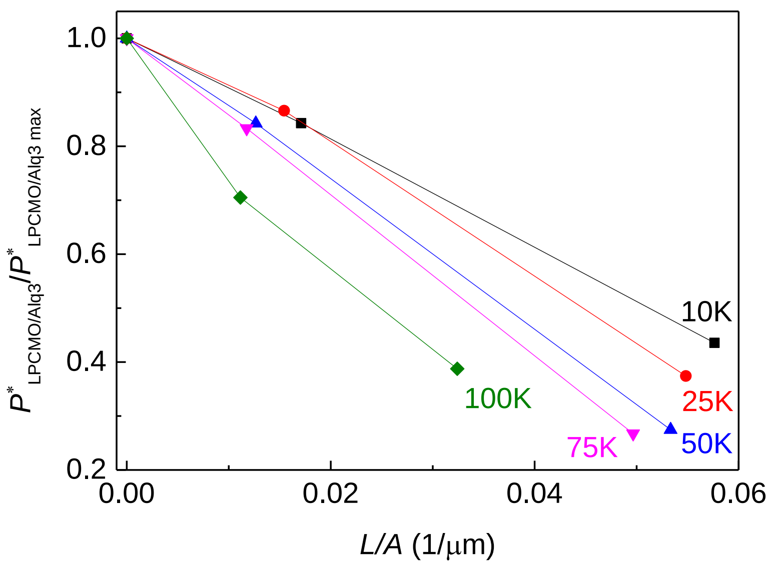

Supplementary Figure 13. The normalized effective spin polarization of the LPCMO/Alq$_3$ spinterface as a function of phase boundary length per FMM area at different temperatures. Calculated $P^*_{\mathrm{LPCMO/Alq3}}/P^*_{\mathrm{LPCMO/Alq3\ max}}$ using the Jullière formula as a function of *L/A* ratio, MR measurements were taken at $V = 0.2$V.

Supplementary Note 1. The estimated spin polarization of the LPCMO thin film

To our best knowledge, there is no literature report on the spin polarization of the phase-separated LPCMO system. We can estimate the spin polarization of LPCMO from the TMR value of the LPCMO/Al_2O_3/Co devices (as shown in Supplementary Fig. 1) using the Jullière model[9],

$$TMR = \frac{2P_{\mathrm{LPCMO}}P_{\mathrm{Co}}}{1-P_{\mathrm{LPCMO}}P_{\mathrm{Co}}} \tag{1}$$

The largest TMR that we measured is 93% at 10 K. Using P_{Co} ~ 34%[10], we obtain P_{LPCMO} ~ 93% at 10 K.

Supplementary Note 2. Connection to the Jullière model

Here we show that our modified two-current model reduces to the Jullière model under further approximations to the tunnel junction, and that these additional approximations are not appropriate for organic spin valves with spinterfaces.

The derivation of the Jullière model starts from assuming that the junction resistance dominates the overall device resistance and neglecting the resistance of the ferromagnetic electrodes. It also neglects the spin-flip scattering. Thus one must set $R_{sf} = \infty$, and $R_{LPCMO\uparrow} = R_{LPCMO\downarrow} = R_{Co\uparrow} = R_{Co\downarrow} = R_{Alq3} = 0$. The next approximation is to set the thickness of Alq3 to zero, thus merging the two spinterfaces into one. The resistances for the parallel configuration are,

$$R_1 = R_3 = 0 \tag{2}$$

$$R_2 = R_{\mathrm{spinter}\uparrow\uparrow} \tag{3}$$

$$R_4 = R_{\mathrm{spinter}\downarrow\downarrow} \tag{4}$$

The total parallel resistance is,

$$R_P = \frac{(R_1+R_2)(R_3+R_4)}{R_1+R_2+R_3+R_4} = \frac{R_{\mathrm{spinter}\uparrow\uparrow}R_{\mathrm{spinter}\downarrow\downarrow}}{R_{\mathrm{spinter}\uparrow\uparrow}+R_{\mathrm{spinter}\downarrow\downarrow}} \tag{5}$$

The resistances for the antiparallel configuration are,

$$R_1 = R_3 = 0 \tag{6}$$

$$R_2 = R_{\mathrm{spinter}\uparrow\downarrow} \tag{7}$$

$$R_4 = R_{\mathrm{spinter}\downarrow\uparrow} \tag{8}$$

The total antiparallel resistance is,

$$R_{AP} = \frac{(R_1+R_2)(R_3+R_4)}{R_1+R_2+R_3+R_4} = \frac{R_{\mathrm{spinter}\uparrow\downarrow}R_{\mathrm{spinter}\downarrow\uparrow}}{R_{\mathrm{spinter}\uparrow\downarrow}+R_{\mathrm{spinter}\downarrow\uparrow}} \tag{9}$$

The last approximation needed to derive the Jullière model is that the spin-dependent junction resistance depends only on the spin-dependent density of states at the Fermi energy of the electrodes. Using a spin-independent constant prefactor g,

$$\frac{1}{R_{\mathrm{spinter}\uparrow\uparrow}} = g n_{\mathrm{L}\uparrow} n_{\mathrm{R}\uparrow} \tag{10}$$

$$\frac{1}{R_{\mathrm{spinter}\downarrow\downarrow}} = g n_{\mathrm{L}\downarrow} n_{\mathrm{R}\downarrow} \tag{11}$$

$$\frac{1}{R_{\mathrm{spinter}\uparrow\downarrow}} = g n_{\mathrm{L}\uparrow} n_{\mathrm{R}\downarrow} \tag{12}$$

$$\frac{1}{R_{\mathrm{spinter}\downarrow\uparrow}} = g n_{\mathrm{L}\downarrow} n_{\mathrm{R}\uparrow} \tag{13}$$

The MR is expressed by,

$$MR = \frac{R_{\mathrm{AP}}}{R_{\mathrm{P}}} - 1 = \frac{n_{\mathrm{L}\uparrow}n_{\mathrm{R}\uparrow}+n_{\mathrm{L}\downarrow}n_{\mathrm{R}\downarrow}-n_{\mathrm{L}\uparrow}n_{\mathrm{R}\downarrow}-n_{\mathrm{L}\downarrow}n_{\mathrm{R}\uparrow}}{n_{\mathrm{L}\uparrow}n_{\mathrm{R}\downarrow}+n_{\mathrm{L}\downarrow}n_{\mathrm{R}\uparrow}} = \frac{2P_{\mathrm{L}}P_{\mathrm{R}}}{1-P_{\mathrm{L}}P_{\mathrm{R}}} \tag{14}$$

where

$$P_{\mathrm{L}} = \frac{n_{\mathrm{L}\uparrow}-n_{\mathrm{L}\downarrow}}{n_{\mathrm{L}\uparrow}+n_{\mathrm{L}\downarrow}} \tag{15}$$

And

$$P_{\mathrm{R}} = \frac{n_{\mathrm{R}\uparrow}-n_{\mathrm{R}\downarrow}}{n_{\mathrm{R}\uparrow}+n_{\mathrm{R}\downarrow}} \tag{16}$$

It is clear from this derivation that the Jullière model is a special case of Fert's two-current model with the additional approximations that neglect all of the ferromagnetic electrode resistances and the spinterface effects, in particular the spin filtering effect.

One can force the spin filtering effect into the Jullière formula by assuming effective spin polarization parameters that are now dependent on the spacer layer. Following the practice of previous works[1,9], we use $P^{*}_{\mathrm{Alq3/Co}}$ and $P^{*}_{\mathrm{LPCMO/Alq3}}$ to represent the effective interfacial spin polarization arising from the Alq3/Co and LPCMO/Alq3 spinterfaces, respectively[11,12]. We apply the Jullière formula in the limit of infinite spin diffusion through the organic medium (i.e., $d \ll \lambda_{\mathrm{sf}}$, where d and λ_{sf} are the thickness and spin diffusion length of the Alq3 spacer layer, respectively). Considering the fact that $P^{*}_{\mathrm{Alq3/Co}}$ is a field independent constant, the change of $P^{*}_{\mathrm{LPCMO/Alq3}}$ will be solely responsible for the tunable MR response in the LPCMO-OSV device. In Supplementary Fig. 13, we plot $P^{*}_{\mathrm{LPCMO/Alq3}}/P^{*}_{\mathrm{LPCMO/Alq3\ max}}$ as a function of L/A. One expects that all data to fall on a single curve. Instead, we see large differences between different temperatures, with the ratio dropping more rapidly as the temperature is increased. It suggests that the effective polarization parameter does not capture all the key physics related to the observed tunable MR.

Supplementary Note 3. Two-current model with spin-flip scattering

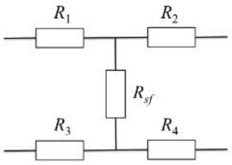

In the LPCMO/Alq3/Co device, we need to consider spin-flip scattering in LPCMO due to spin-orbit coupling. This is strongest at the phase boundary (and the interface) because of broken symmetry. Therefore, the spin-flip scattering rate is approximately proportional to the total area of the phase boundary. To account for the spin-flip scattering, we modify Fert's two-current model by adding an effective resistance due to spin-flip scattering, as shown in the figure. R_{sf} should be inversely proportional to the area of the phase boundary. This modified two-current model is solved as the following.

$$I_1R_1 + I_2R_2 = V \tag{17}$$

$$I_3R_3 + I_4R_4 = I_1R_1 + I_2R_2 \tag{18}$$

$$I_1 + I_3 = I_2 + I_4 \tag{19}$$

$$I_1R_1 + (I_1 - I_2)R_{\mathrm{sf}} = I_3R_3 \tag{20}$$

The total resistance is found as

$$R = \frac{(R_2+R_4)R_1R_3+(R_1+R_3)R_2R_4+R_{sf}(R_1+R_2)(R_3+R_4)}{(R_1+R_3)(R_2+R_4)+R_{\mathrm{sf}}(R_1+R_2+R_3+R_4)} \tag{21}$$

Now we apply the modified two-current model to the OSV for each spin configuration. For parallel moment alignment, the resistances in the up-spin channel are

$$R_1 = R_{\mathrm{LPCMO\uparrow}} \tag{22}$$

$$R_2 = R_{\mathrm{Co\uparrow}} + R_{\mathrm{Alq3}} + R_{\mathrm{spinterL\uparrow}} + R_{\mathrm{spinterR\uparrow}} \tag{23}$$

where R_{Alq3} is the spin-independent part of the junction resistance and $R_{\mathrm{spinterL\uparrow}}$ and $R_{\mathrm{spinterR\uparrow}}$ are the left (LPCMO spinterface) and right (Co spinterface) spin-dependent resistance of the spinterfaces. The resistances in the down spin channel are,

$$R_3 = R_{\mathrm{LPCMO\downarrow}} \tag{24}$$

$$R_4 = R_{\mathrm{Co\downarrow}} + R_{\mathrm{Alq3}} + R_{\mathrm{spinterL\downarrow}} + R_{\mathrm{spinterR\downarrow}} = \infty \tag{25}$$

Here we assume that the parallel down-spin channel resistance is very large compared to all other resistance. Assuming a finite parallel down-spin resistance will not be able to reproduce the large experimental increase in the MR with a preset magnetic field is applied. For antiparallel moment alignment, the resistances are

$$R_1 = R_{\mathrm{LPCMO\downarrow}} \tag{26}$$

$$R_2 = R_{\mathrm{Co\uparrow}} + R_{\mathrm{Alq3}} + R_{\mathrm{spinterL\downarrow}} + R_{\mathrm{spinterR\uparrow}} \tag{27}$$

$$R_3 = R_{\mathrm{LPCMO\uparrow}} \tag{28}$$

$$R_4 = R_{\mathrm{Co\downarrow}} + R_{\mathrm{Alq3}} + R_{\mathrm{spinterL\uparrow}} + R_{\mathrm{spinterR\downarrow}} \tag{29}$$

To facilitate solving the equations, we assume large spin-filter effect on both spinterfaces, thus neglect all up-spin channel spinterface resistances. We then define the ratios,

$$\lambda_{\mathrm{L}} = \frac{R_{\mathrm{spinterL\downarrow}}}{R_{\mathrm{Alq3}}},\ \lambda_R = \frac{R_{\mathrm{spinterR\downarrow}}+R_{\mathrm{Co\downarrow}}}{R_{\mathrm{Alq3}}} \tag{30}$$

The equations are rewritten in terms of λ_L and λ_R,

$$R_{\mathrm{AP}} = \frac{(1+\lambda_{\mathrm{L}})R_{\mathrm{Alq3}}+R_{\downarrow}}{(2+\lambda_{\mathrm{L}}+\lambda_{\mathrm{R}})R_{\mathrm{Alq3}}+R_{\downarrow}}(1+\lambda_{\mathrm{R}})R_{\mathrm{Alq3}} \tag{31}$$

where an effective LPCMO down-spin resistance is defined as,

$$R_{\downarrow}=\frac{R_{sf}R_{LPCMO\downarrow}}{R_{sf}+R_{LPCMO\downarrow}} \tag{32}$$

Solve for $R_{\downarrow}$ from the equation,

$$R_{\downarrow}=\frac{(2+\lambda_L+\lambda_R)R_{AP}-(1+\lambda_L)(1+\lambda_R)R_{Alq3}}{(1+\lambda_R)R_{Alq3}-R_{AP}}R_{Alq3}=\frac{(2+\lambda_L+\lambda_R)R_{AP}-(1+\lambda_L)(1+\lambda_R)R_P}{(1+\lambda_R)R_P-R_{AP}}R_P \tag{33}$$

Because $R_{\downarrow}>0$, we must have,

$$R_{AP}<(\lambda_R+1)R_P \tag{34}$$

and

$$(1+\lambda_L)R_P<R_{AP} \tag{35}$$

Over all preset magnetic fields,

$$\lambda_L<\min\left(\frac{R_{AP}}{R_P}\right)-1 \tag{36}$$

$$\lambda_R>\max\left(\frac{R_{AP}}{R_P}\right)-1 \tag{37}$$

At the smallest preset field, $R_{\downarrow}\approx 0$, so

$$1+\lambda_R\approx\frac{(1+\lambda_L)\left[\min\left(\frac{R_{AP}}{R_P}\right)-1\right]}{\min\left(\frac{R_{AP}}{R_P}\right)-(1+\lambda_L)}>\max\left(\frac{R_{AP}}{R_P}\right) \tag{38}$$

Thus

$$\lambda_L>\frac{\min\left(\frac{R_{AP}}{R_P}\right)\max\left(\frac{R_{AP}}{R_P}\right)}{\max\left(\frac{R_{AP}}{R_P}\right)+\min\left(\frac{R_{AP}}{R_P}\right)-1}-1 \tag{39}$$

We take λ_L to be at the midpoint of its two limits,

$$\lambda_L=\frac{1}{2}\min\left(\frac{R_{AP}}{R_P}\right)\left[1+\frac{\max\left(\frac{R_{AP}}{R_P}\right)}{\max\left(\frac{R_{AP}}{R_P}\right)+\min\left(\frac{R_{AP}}{R_P}\right)-1}\right]-1 \tag{40}$$

Then,

$$\lambda_R=\frac{(1+\lambda_L)\left[\min\left(\frac{R_{AP}}{R_P}\right)-1\right]}{\min\left(\frac{R_{AP}}{R_P}\right)-(1+\lambda_L)}-1=2\max\left(\frac{R_{AP}}{R_P}\right)+\min\left(\frac{R_{AP}}{R_P}\right)-2 \tag{41}$$

Once λ's are determined, we can use them to find all other resistances under any field.

For LPCMO/Al_2O_3/Co devices, there are no spinterfaces. In this case, the resistances for the parallel configuration are,

$$R_1=R_{LPCMO\uparrow} \tag{42}$$

$$R_2=R_{Co\uparrow}+R_{Al2O3} \tag{43}$$

where R_{Al2O3} is the spin-independent part of the junction resistance.

$$R_3=R_{LPCMO\downarrow} \tag{44}$$

$$R_4=R_{Co\downarrow}+R_{Al2O3} \tag{45}$$

And for the antiparallel configuration, the resistances are

$$R_1=R_{LPCMO\downarrow} \tag{46}$$

$$R_2=R_{Co\uparrow}+R_{Al2O3} \tag{47}$$

$$R_3=R_{LPCMO\uparrow} \tag{48}$$

$$R_4=R_{Co\downarrow}+R_{Al2O3} \tag{49}$$

Assuming that the Co resistance and the up-spin LPCMO resistance are small,

$$R_P=\frac{R_{LPCMO\downarrow}R_{Al2O3}+R_{sf}(R_{LPCMO\downarrow}+R_{Al2O3})}{2R_{LPCMO\downarrow}R_{Al2O3}+R_{sf}(2R_{Al2O3}+R_{LPCMO\downarrow})}R_{Al2O3} \tag{50}$$

$$R_{AP}=\frac{R_{LPCMO\downarrow}R_{Al2O3}+R_{sf}(R_{LPCMO\downarrow}+R_{Al2O3})}{R_{LPCMO\downarrow}(R_{Co\downarrow}+2R_{Al2O3})+R_{sf}(R_{LPCMO\downarrow}+R_{Co\downarrow}+2R_{Al2O3})}(R_{Co\downarrow}+R_{Al2O3}) \tag{51}$$

A small MR approximately equal to $R_{\mathrm{Co}\downarrow}/R_{\mathrm{Al2O3}}$, if one neglects the electrode resistance, is predicted,

$$MR = \frac{R_{\mathrm{AP}}}{R_{\mathrm{P}}} - 1 = \frac{R_{\mathrm{Co}\downarrow}}{R_{\mathrm{Al2O3}}} - \left(1 + \frac{R_{\mathrm{Co}\downarrow}}{R_{\mathrm{Al2O3}}}\right) \frac{R_{\mathrm{Co}\downarrow}}{R_{\mathrm{Co}\downarrow} + 2R_{\mathrm{Al2O3}} + R_{\downarrow}} \quad (52)$$

The extra term on the righthand side shows that in the absence of the spin filter effect, electrode resistance tends to reduce the MR. On the other hand, although we assume that all of $R_{\downarrow}$, $R_{\mathrm{Co}\downarrow}$, and R_{Al2O3} scale linearly with the effective conducting cross section of the junction when the preset field is applied, $R_{\downarrow}$ should change slightly faster than the other resistances since it is at the source of the change. This in turn causes a slight reduction of MR for the Al_2O_3 junction with increasing B_{pre}, as observed experimentally.

Supplementary References

1. Xiong, Z. H., Wu, D., Vardeny, Z. V., & Shi, J. Giant magnetoresistance in organic spin-valves. *Nature* **427,** 821-824 (2004).
2. Barraud, Clément. et al. Unravelling the role of the interface for spin injection into organic semiconductors. *Nature Physics*. **6,** 615-620 (2010)
3. Sun, D. et al. Giant magnetoresistance in organic spin valves. *Physical Review Letters*. **104,** 236602 (2010).
4. Wang, F. J., Yang, C. G., Vardeny, Z. V., & Li, X. G. Spin response in organic spin valves based on $La_{2/3}Sr_{1/3}MnO_3$ electrodes. *Physical Review B* **75,** 245324 (2007).
5. Dediu, V., et al. Room-temperature spintronic effects in Alq_3-based hybrid devices. *Physical Review B* **78,** 115203 (2008).
6. Singh-Bhalla, G. et al. Intrinsic tunneling in phase separated manganites. *Physical Review Letters*. **102,** 077205 (2009).
7. Gould, C. et al. Tunneling anisotropic magnetoresistance: a spin-valve-like tunnel magnetoresistance using a single magnetic layer. *Physical Review Letters*. **93,** 117203 (2004).
8. Singh-Bhalla, G., Biswas, A., & Hebard, A. F. Tunneling magnetoresistance in phase-separated manganite nanobridges. *Physical Review B* **80,** 144410 (2009).
9. Julliere, M. Tunneling between ferromagnetic films. *Physics Letters A*. **54,** 225-226 (1975).
10. Tedrow, P. M., & Meservey, R. Spin polarization of electrons tunneling from films of Fe, Co, Ni, and Gd. *Physical Review B* **7,** 318 (1973).
11. Sanvito, S. Molecular spintronics: the rise of spinterface science. *Nature Physics*. **6,** 562-564 (2010).
12. Cinchetti, M., Dediu, V. A., & Hueso, L. E. Activating the molecular spinterface. *Nature Materials*. **16,** 507 (2017).
